# Telerehabilitation for COPD patients across sectors: using technology to promote community of practice among healthcare professionals

**Published:** 2012-06-15

**Authors:** Birthe Dinesen

**Affiliations:** Aalborg University, Denmark

**Keywords:** telerehabilitation, co-operation, co-ordination, activities, knowledge-sharing

## Abstract

**Introduction:**

The TELEKAT project (“Telehomecare, chronic patients and the integrated healthcare system”) has developed a telerehabilitation programme enabling patients with chronic obstructive pulmonary disease (COPD) to perform preventive home monitoring across sectors. The goal of the project is to help patients avoid readmission to hospital, perform self-monitoring and to maintain rehabilitation activities in their homes. The study includes COPD patients with severe and very severe COPD (n=111). Nurses and doctors at a hospital, GPs, district nurses and nurses from a healthcare center have monitored COPD patients in the programme.

**Aim:**

The aim of this paper is to discuss how telerehabilitation technology affects interaction between healthcare professionals in the Telekat network.

**Theory:**

Inter-organisational theory and learning theory.

**Methods:**

The case study approach was applied using a triangulation of data collection techniques. The data included documents, participant-observation (163 hours) and qualitative interviews with nurses and doctors at a hospital, GPs, district nurses, nurses and physiotherapists at a healthcare centre (n=40). All the interviews were transcribed and data were coded using Nvivo 8.0 software, as inspired by Kvale (2009).

**Results:**

The healthcare professionals expressed the view that during the process of creating and testing the programme for telerehabilitation, they have created new roles in the cooperation between district nurses, GPs, nurses and doctors at hospital and healthcare centre. The roles have changed concerning counseling on rehabilitation issues. Through the process of developing the programme of telerehabilitation, mutual trust has emerged between the healthcare professionals, enhancing collaboration and coordination activities related to telerehabilitation of COPD patients across sectors. Video conferences between the healthcare professionals have facilitated knowledge-sharing and reflections on professionals’ issues between experts and generalists. Finally, the healthcare professionals expressed the view that participation and interaction via the technology allows them to feel part of a community of telerehabilitation.

**Conclusion:**

The telerehabilitation technology facilitates participation and interaction between the healthcare professionals across sectors, giving them a feeling of belonging to a part of a community of telerehabilitation.

